# Efficacy, toxicity and histopathological analysis of *Astrocaryum aculeatum* oil solid lipid nanoparticles on *Colossoma macropomum* infected by parasitic dactylogyrideans

**DOI:** 10.1590/S1984-29612026016

**Published:** 2026-05-25

**Authors:** Bruna David Brito, Marcela Nunes Videira, Abthyllane Amaral de Carvalho, Maiara Ferreira Martins, Victor Hugo Souza Marinho, Irlon Maciel Ferreira, Eliane Tie Oba Yoshioka, Marcos Tavares-Dias

**Affiliations:** 1 Universidade Federal do Amapá - UNIFAP, Programa de Pós-graduação em Biodiversidade Tropical - PPGBio, Macapá, AP, Brasil; 2 Universidade do Estado do Amapá - UEAP, Laboratório de Morfofisiologia e Sanidade Animal, Macapá, AP, Brasil; 3 Universidade Federal do Amapá - UNIFAP, Laboratório de Biocatálise e Síntese Orgânica Aplicada, Macapá, AP, Brasil; 4 Empresa Brasileira de Pesquisa Agropecuária - Embrapa Amapá, Macapá, AP, Brasil

**Keywords:** Freshwater fish, Monogenea, nanoparticles, parasite, treatment, tucumã, Peixe de água doce, Monogenea, parasito, tratamento, tucumã

## Abstract

This study investigated the efficacy of *Astrocaryum aculeatum* fixed oil solid lipid nanoparticles (SLNs) on dactylogyridean parasites of *Colossoma macropomum* gills and effects on hematology and gill histology. The main fatty acid component of *A. aculeatum* oil was oleic acid (68.0%). Among the concentrations of *A. aculeatum* fixed oil SLNs tested (250-1000 mg/L), the fish tolerated only 250 mg/L, which was the concentration used in the consecutive five baths of 1 h per day. Two control groups were also used: one with myristic acid + Tween 80 and another with only water from the cultivation tank. The baths have no efficacy against *Anacanthorus spathulatus*, *Notozothecium janauachensis*, and *Mymarothecium boegeri* from *C. macropomum* gills. Detachment of the gill lamellar epithelium, lamellar epithelial hyperplasia, lamellar hyperplasia with fusion, and aneurysm were damages caused by parasites and not by the treatments. Total number of lymphocytes decreased in the fish exposed to baths with 250 mg/L of *A. aculeatum* fixed oil SLNs, while other blood parameters were not affected. Although the treatments with *A. aculeatum* fixed oil SLNs were not effective against parasitic dactylogyrideans as expected, they were not toxic for C*. macropomum*, because they did not provoke alterations in gills and in physiology.

## Resumo

Foi investigada a eficácia de nanopartículas lipídicas sólidas (NLSs) de óleo fixo de *Astrocaryum aculeatum* em parasitos dactilogirídeos nas brânquias de *Colossoma macropomum* e seus efeitos na hematologia e histopatologia. O principal componente de ácido graxo do óleo fixo de *A. aculeatum* foi o ácido oleico (68,0%). Entre as concentrações de NLSs de óleo de *A. aculeatum* testadas (250-1000 mg/L), os peixes toleraram somente 250 mg/L, que foi a concentração utilizada nos cinco banhos consecutivos de 1 hora por dia. Dois grupos controles foram usados: um com ácido mirístico + Tween 80 e outro com água do tanque de cultivo. Banhos com 250 mg/L de NLSs de óleo fixo de *A. aculeatum* não tiveram eficácia contra *Anacanthorus spathulatus*, *Notozothecium janauachensis* e *Mymarothecium boegeri*. Descolamento do epitélio lamelar branquial, hiperplasia epitelial lamelar, hiperplasia lamelar com fusão e aneurisma foram danos causados ​​pelos parasitas. O número total de linfócitos diminuiu nos peixes expostos a 250 mg/L de NLSs de óleo fixo de *A. aculeatum*, enquanto outros parâmetros sanguíneos não foram afetados. Embora os tratamentos com NLSs de óleo fixo de *A. aculeatum* não tenham sido eficazes contra dactilogirídeos como esperado, eles não foram tóxicos, pois não provocaram alterações e nas brânquias e fisiologia dos peixes.

## Introduction

Aquaculture plays a significant role in the global economy, ensuring food security and generating employment. This major food production industry provides a source of high-quality protein for the growing global population. Fish are an important source of income and livelihood for billions of people around the world, many of whom depend on fish protein for nutrition (FAO, 2024; Ahmed et al., 2024; Kumari et al., 2024; Brito et al., 2026). However, intensive practices in fish aquaculture have led to serious environmental and management problems, including disease outbreaks that threaten this important global industry. Parasites have caused tremendous negative economic impacts for global aquaculture, with estimated annual losses from USD 1.05 billion to 9.58 billion (Shinn et al., 2015). Therefore, controlling parasites in fish aquaculture with effective therapeutic products is crucial to improve production and productivity (Wang et al., 2008; Malheiros et al., 2023, 2025; Qu et al., 2023; Kumari et al., 2024).

Parasitic diseases by Monopisthocotyla and Polyopisthocotyla species have drastically affected the production of the fish aquaculture industry (Wang et al., 2008; Tavares-Dias & Martins, 2017; Kumari et al., 2024; Sajiri et al., 2025; Malheiros et al., 2025; Brito et al., 2026). Monopisthocotyleans primarily feed on epithelial tissues, whereas polyopisthocotyleans feed on blood of the host fish (Sajiri et al., 2025), while both usually have direct life cycle and with short generation time. Dactylogyrideans infections, frequently caused by *Anacanthorus spathulatus* Kritsky, Thatcher & Kayton 1979, *Notozothecium janauachensis* Belmont-Jégui, Domingues & Martins 2004, and *Mymarothecium boegeri* Kritsky, Thatcher & Kayton 1979 (Monopisthocotyla: Dactylogyridae), have also affected the health, growth, and overall productivity of tambaqui *Colossoma macropomum* (Cuvier, 1816). Consequently, tambaqui-dactylogyrideans system is an excellent model for evaluating novel drugs delivery technologies (e.g., solid lipid nanoparticles), given the poor water solubility of fixed oils.

In fish aquaculture, the indiscriminate use of large quantities of chemotherapeutants (e.g., formalin, organophosphates, praziquantel, albendazole, and ivermectin) has led to the development of resistance in parasites and accumulation of residues in fish muscles (Malheiros et al., 2023; Kumari et al., 2024; Brito et al., 2026), which may cause a grave problem in the health consumer. In addition, some are ineffective, negatively impact water quality, and are toxic to host fish, environment and humans (Malheiros et al., 2022, 2023; Kumari et al., 2024; Brito et al., 2026). For these reasons, the use of some chemicals products has been restricted to aquaculture in many countries. Effective and environmentally friendly methods for controlling parasites and diseases in fish aquaculture require the development of new treatments compared to these usuals chemotherapeutics that have been used for a long time (Valentim et al., 2018; Tavares-Dias, 2018; Malheiros et al., 2023, 2025; Qu et al., 2023; Brito et al., 2026). Phytotherapy has emerged as a promising alternative due to the presence of bioactive compounds in medicinal plants that have therapeutic potential. For the control of parasitic dactylogyrideans infecting *C. macropomum*, *Copaifera reticulata* oleoresin (Malheiros et al., 2022), *Carapa guianensis* fixed oil (Malheiros et al., 2023), solid lipid nanoparticles of *Pentaclethra macroloba* oil (Malheiros et al., 2025) and *Piper hispidum* essential oil (Alves et al., 2024) have been used. Various other studies have emphasized the importance of using oils or their major constituents for therapeutic purposes (Jobim et al., 2014; Tavares-Dias, 2018; Valentim et al., 2018; Malheiros et al., 2023; Qu et al., 2023; Alves et al., 2024; Brito et al., 2026), including fixed oils, which are insoluble in water. Consequently, only few fixed oils (Malheiros et al., 2022, 2023; Brito et al., 2026) have been used to control parasite infections in fish aquaculture. This insolubility of fixed oils results in low bioaccessibility and bioavailability of their bioactive compounds. It has been suggested that this water insolubility of different oils can be resolved using nanotechnology methods, such as the use of solid lipid nanoparticles (SLNs) (Qu et al., 2023; Mogahed et al., 2023; Abdelkarim et al., 2025). SLNs are colloidal particles (50-1000 nm) made from solid lipids at room temperature and containing surfactants as stabilizers or emulsifiers. SLNs are a drug delivery system with low toxicity, good biocompatibility, high bioavailability, and physical stability. They are also physiologically compatible, biodegradable, and easy to produce on a large scale, like nanoemulsions (Wissing et al., 2004; Nemati et al., 2024; Abdelkarim et al., 2025).

Fixed oil is extracted from *Astrocaryum aculeatum* (Arecaceae) fruit at room temperature (Ibiapina et al., 2022), a tropical palm popularly known as tucumã, and this is widely distributed into Brazilian Amazon and parts of Central America. The *A. aculeatum* oil has a lipid composition that includes mono- and polyunsaturated fatty acids, carotenoids (e.g., β-carotene), phenolic compounds, and phytosterols (Yuyama et al., 2008; Jobim et al., 2014; Linhares et al., 2017; Ibiapina et al., 2022; Machado et al., 2022). Pharmacokinetic studies indicated a high bioavailability of *A. aculeatum* oil bioactive compounds with potential relevance therapeutic, since this exhibits bactericidal, antifungal, anti-inflammatory, antidyslipidemic, antihyperglycemic, cytoprotective, genoprotective, antiproliferative, anticarcinogenic, and neuroprotective activities (Jobim et al., 2014; Machado et al., 2022). However, studies investigating its antiparasitic activity are lacking. Thus, this study investigated the anti- dactylogyridean efficacy of baths with 250 mg/L of *A. aculeatum* oil SLNs, as well as their effects on hematology and histopathology of *C. macropomum* gills.

## Material and methods

### Obtaining *A. aculeatum* fixed oil

Fruits of *A. aculeatum* were collected in the Vale Verde locality, municipality of Macapá, state of Amapá, Brazil, and used to extract oil using a hydraulic press. A total of 22 kg of *A. aculeatum* fruit was used for the extraction of oil. After removing the fruits from the bunch, they were sanitized with running water and sorted. The fruits were pulped, yielding 7.465 kg of wet pulp. This mass was dried in an air circulation oven (Fabbe Ltda., São Paulo, Brazil) at 60ºC for 24 h to remove 95% of the pulp's moisture. After dehydration, 5.8 kg of dry pulp remained. This pulp was cold pressed without solvents using a hydraulic press (Siwa, Atibaia, Brazil) at Embrapa Amapá, state of Amapá, Brazil. The extracted fixed oil was filtered using a Büchner filter. A total yield of 83.3% *A. aculeatum* oil was obtained. The physicochemical properties of the *A. aculeatum* fixed oil, such as the acidity and saponification indices, density, and peroxide content, were determined according to the methods described in the analytical standards of the Adolfo Lutz Institute (IAL, 2008).

### Chemical composition and nuclear magnetic resonance (NMR) ^13^C analyses, and preparation of fatty acid ethyl esters from *A. aculeatum* fixed oil

The fatty acid ethyl esters (FAEE) samples were prepared from *A. aculeatum* fixed oil through a transesterification reaction. This reaction was carried out in a 25 mL flask containing 1.0 g of fixed oil, 4 mL of ethanol, and 0.1 g of Oxone^®^ (150%), according to the methodology described by Lopes et al. (2021). The reaction mixture was stirred at 40 °C for 12 hours, and the formed product was monitored by thin-layer chromatography. The mixture was then filtered and dried with anhydrous sodium sulfate. Finally, the mixture was purified by flash chromatography using silica gel as the stationary phase and n-hexane acetate (9:1) as the eluent.

Samples of FAEE from *A. aculeatum* fixed oil were analyzed using gas chromatography coupled with *gas chromatography-mass spectrometry* (GC-MS). The analyses were performed using a Shimadzu GC system equipped with a mass selective detector (Shimadzu, MS2010 plus, Japan) in electron ionization mode (EI, 70 eV) and equipped with a 30 m × 0.25 mm × 0.25 μm RTX-5MS column. The GC-MS conditions were as follows: the oven started at 130 °C, remained at this temperature for 2 min, increased to 290 °C at a rate of 5 °C per minute, and was held at this temperature for 2 min. The total analysis time was 36 min. The injector and detector temperatures were maintained at 210 °C; 1 μL of the sample was injected with a 1:15 split ratio. Helium was used as the carrier gas at a flow rate of 1.0 mL/min. Ions were monitored from 3 to 36 min at *m/z* 40–500. The components present in the samples were identified by comparing the spectral data with the data in the Wiley library (Marinho et al., 2023).

The ^13^C nuclear magnetic resonance analyses were performed on a 400 MHz Premium Shielded spectrometer from Agilent Technologies. The sample was prepared from 20 μL of *A. aculeatum* oil, which was solubilized in 600 μL of deuterated chloroform (CDCl3, Cambridge Isotope Laboratories, Andover, USA) with tetramethylsilane (TMS) serving as the internal standard. Chemical deviations were expressed in mg/L (Sarquis et al., 2020).

### Preparation of SLNs with *A. aculeatum* fixed oil

The *A. aculeatum* oil SLNs were prepared according to the methodology described by Oliveira et al. (2022), with the following modifications. The aqueous phase consisted of the surfactant Tween 80 (5% by weight) dissolved in distilled water (89% by weight). The lipid phase consisted of the co-surfactant myristic acid (1% by weight), which was dissolved in *A. aculeatum* oil (5% by weight) at the desired concentrations and heated to 65 °C for 45 min. The lipid and aqueous phases were then mixed and stirred in a vortex for 10 min. The final concentrations of *A. aculeatum* oil were prepared 24 h before use.

Myristic acid (99%), Tween 80^®^ (polyoxyethylene sorbitan monooleate, HLB 15.0), methanol, and ethanol were purchased from Sigma-Aldrich (São Paulo, Brazil).

### Analyses of particle size, polydispersity index, and zeta potential of the *A. aculeatum* fixed oil SLNs

The droplet size, polydispersity index (PDI), and zeta potential of SLNs with *A. aculeatum* fixed oil were determined using a Zetasizer Nano ZS (Malvern, United Kingdom). Each sample was diluted with distilled water (1:10) to avoid multiple scattering effects, in accordance with Oliveira et al. (2022). All analyses were performed at 25 °C. Measurements were taken in triplicate, and the mean droplet size was expressed as the mean diameter.

#### Fish, acclimation, and dactylogyridean parasites

Fingerlings of *C. macropomum* were purchased from a commercial fish farm and were transported to the Aquaculture and Fisheries Laboratory at Embrapa Amapá (Macapá, state of Amapá, Brazil). For 10 days, the fish were acclimated in a 500-L water tank with constant water renewal and aeration. The following water quality parameters were maintained: temperature 30.3 ± 0.1 °C, dissolved oxygen 5.5 ± 0.2 mg/L, pH 5.4 ± 0.2, total ammonia 0.05 ± 0.2 mg/L, alkalinity 10.0 ± 0.001 mg/L, and hardness 10.0 ± 0.001 mg/L. These parameters were measured every two days using a multi-parameter probe (Horiba, model U-52, Japan).

During the acclimation period, the fish were fed a diet containing 32% crude protein (CP) four times a day. Before the start of the bath treatments, 20 fish were euthanized and examined for the presence of dactylogyrideans in the gills. All fish examined were found to be parasitized (mean abundance of 22 ± 15).

#### Tolerance test of *C. macropomum* to exposure with *A. aculeatum* oil SLNs

Tolerance tests were carried out to assess the toxicity of *A. aculeatum* fixed oil SLNs for *C. macropomum*, aiming to determine the supported concentration by fish, for use in safe therapeutic baths. A total of 45 fish (32.9 ± 8.7 g and 13.1 ± 1.1 cm) were used for these trials and were distributed into three 100-L tanks, with five fish in each replicate. Different concentrations of SLNs with *A. aculeatum* fixed oil were used: 250, 500, and 1000 mg/L. The tolerance tests with these concentrations of *A. aculeatum* fixed oil lasted 8 h, during which the time of mortality and the behavior of the fish were observed and recorded. Fish mortality was determined when they ceased the opercular movements and tail beating and no longer responded to mechanical stimuli.

#### Therapeutic baths with *A. aculeatum* fixed oil SLNs on *C. macropomum*

Based on the results of the tolerance tests, therapeutic baths were conducted with 117 *C. macropomum* specimens (32.9 ± 8.7 g and 13.1 ± 1.1 cm), which were randomly distributed in 100-L water tanks. The naturally parasitized fish were kept in a running water system with aeration until *A. aculeatum* oil SLNs were added. At this point, this running water system was turned off to maintain only aeration during the baths. The experimental design of the baths consisted of three treatments with three replicates each, containing 13 fish per replicate (39 fish per treatment). Two controls were included: one with only water from the cultivation tank and the other with myristic acid + Tween 80. Each bath lasted 1 hour for five consecutive days. After the final bath, the fish were euthanized by medullary section. The gills of 10 animals from each replicate (30 fish per treatment) were collected and fixed in 5% formalin to quantify and identify the parasite species (Eiras et al., 2006) and determine the prevalence and mean abundance (Bush et al., 1997). The antiparasitic efficacy of the therapeutic baths was determined using the methodology previously described by Wang et al. (2008).

#### Blood parameters of *C. macropomum* after therapeutic baths with *A. aculeatum* fixed oil SLNs

After the therapeutic baths, and before gill removal, one blood sample was collected from the caudal vessel of five *C. macropomum* specimens from all treatments (250 mg/L of *A. aculeatum* fixed oil SLNs, cultivation tank water, and myristic acid + Tween 80) in each of the three replicates (15 fish per treatment) using syringes containing EDTA (10%). The blood was immediately divided into two aliquots. One blood aliquot was used to count erythrocytes with a hemocytometer and determine hematocrit and hemoglobin concentration using the microhematocrit and cyanmethemoglobin methods, respectively. These data were then used to calculate the hematimetric indices: mean corpuscular volume (MCV) and mean corpuscular hemoglobin concentration (MCHC). Blood smears were made in duplicates, stained panchromatically with a combination of May-Grünwald-Giemsa-Wright, and used for leukocyte counting. Leukocyte identification, nomenclature, and total leukocyte and thrombocyte counts were performed according to Ranzani-Paiva et al. (2013). The second blood aliquot was centrifuged at 75 G (Centrifuge MCD-2000, Brazil) for 7 min to obtain plasma and determine the concentrations of glucose and total protein. Glucose concentration was determined using the enzymatic-colorimetric method of glucose oxidase with a commercial kit (Biotécnica, MG, Brazil). The plasma total protein concentration was determined by the biuret method using a commercial kit (Biotécnica, MG, Brazil). These biochemical parameters were read on a digital spectrophotometer (KASVI Model 2022/2025).

#### Histopathology of *C. macropomum* gills after therapeutic baths with *A. aculeatum* fixed oil SLNs

After the therapeutic baths with *A. aculeatum oil SLNs*, the gill arches of three fish from each replicate (9 fish per treatment) were collected for histopathological analysis. The first gill arch from both sides (right and left) was collected, fixed in Davidson's solution for 48 h, and then dehydrated in a series of gradually increasing ethanol (70, 80, 90, and 100%) and xylene. The gill arches were embedded in paraffin, and consecutive 5-μm sections were obtained using a microtome (Easypath EP 31-20,093, Brazil). After making duplicate slides, they were stained with hematoxylin and eosin (HE) and analyzed under optical microscope. Images were taken with a Moticam 2300 3.0 MP digital camera attached to an optical microscope and computer. Histopathological analyses were performed semiquantitatively using the mean assessment values (MAV) according to method by Schwaiger et al. (1997), and the histopathological alteration index (HAI) according to method by Poleksić & Mitrović-Tutundžić (1994).

#### Statistical analysis

All parasitological, hematological, and histopathological data were previously assessed for normality and homoscedasticity using the Shapiro-Wilk and Bartlett tests, respectively. Since these data did not follow a normal distribution, the Kruskal-Wallis test, followed by the Dunn test, was used to compare medians at a 5% probability level (Zar, 2010).

## Results

### Chemical composition of *aculeatum* fixed oil and physicochemical characteristics of the SLNs containing *A. aculeatum* fixed oil

The *A. aculeatum* fixed oil, obtained by cold pressing, was light orange in color and liquid at room temperature (28 ± 3 °C). The acidity index (2.00 ± 0.15 mg/NaOH/g), saponification index (109.69 ±1.00 mg/KOH/g), density (0.989 ± 0.01 g/mL), and peroxide value (0.04 ± 0.01 meq/kg) of *A. aculeatum* fixed oil were evaluated.

The chemical composition of *A. aculeatum* fixed oil identified by gas chromatography-mass spectrometry showed that its fatty acid profile consisted mainly of monounsaturated oleic acid and linoleic acid in low proportion. Additionally, palmitic acid and stearic acid were identified (Table 1). The ^13^C NMR analysis (data not shown) reinforces the presence of abundant unsaturated carbons derived from oleic and linoleic fatty acids through the presence of signals in the ^13^C spectrum between 129 and 130 mg/L. These signals are characteristic of sp2 carbons.

**Table 1:** Ethyl ester composition of *Astrocaryum aculeatum* fixed oil.

**Fatty acid**	**Peak**	**Relative concentration (%)**
Palmitic (C16:0)	1	26.5
Linoleic (C18:2, ʊ-6)	2	2.0
Oleic (C18:1, ʊ-9)	3	68.0
Stearic (C18:0)	4	3.5
Total saturated	-	30.0
Total monounsaturated	-	68.0
Total polyunsaturated	-	2.0

The *A. aculeatum* fixed oil SLNs exhibited a light orange color and were monodisperse, with a particle size of 380.7 ± 1.8 nm, a PDI value of 0.315, and a zeta potential of -43.8 ± 0.7 mV.

### Tolerance of *C. macropomum* to different concentrations of *A. aculeatum* fixed oil SLNs

During the tolerance trials, all fish showed signs of agitation upon initial contact with *A. aculeatum* fixed oil SLNs. At concentrations of 250 and 500 mg/L, some fish exhibited similar behavioral changes, including swimming at the surface, gasping for air, accelerating opercular movement, and descending to the bottom of the tank. At the highest concentration (1,000 mg/L), the fish exhibited all of aforementioned characteristics, as well as jumping out of the water and erratic swimming. Mortality of *C. macropomum* occurred only at concentrations of 500 and 1,000 mg/L of *A. aculeatum* fixed oil SLNs during the 8 h of exposure, while 250 mg/L did not cause mortality and was then defined as the optimal concentration for the therapeutic baths.

### Anti*-*dactylogyrideans efficacy of the therapeutic baths with *A. aculeatum* fixed oil SLNs on *C. macropomum*

During therapeutic baths with 250 mg/L of *A. aculeatum* fixed oil SLNs, some behavioral changes were also observed in the fish, including agitation, accelerated operculum movement, and erratic swimming, similar to observed during tolerance trials. However, the fish recovered once the oil was eliminated from the tanks, and there was no mortality. No behavioral changes were observed in the control groups with water from the cultivation tank or myristic acid + Tween 80. The baths were carried out in the morning, and the fish were fed in the afternoon. Three dactylogyridean species were identified on *C*. *macropomum* gills: *A. spathulatus*, *N. janauachensis*, and *M. boegeri*. These parasites were quantified to determine the therapeutic efficacy of the baths carried out. The prevalence of ectoparasites was higher in the control group fish exposed only to water from the cultivation tank than in fish of the other treatments. The mean abundance of parasites in control group fish with cultivation tank water was higher (p<0.05) than in fish exposed to with myristic acid + Tween 80 and fish treated with 250 mg/L of *A. aculeatum* fixed oil SLNs, which were similar. The treatments with 250 mg/L of *A. aculeatum* fixed oil SLNs were not effective against dactylogyrideans of *C. macropomum* gills, because efficacy of exposure to myristic acid + Tween 80 had high efficacy when compared to control with cultivation tank water (Table 2).

**Table 2:** Parasitological indices of dactylogyrideans on *Colossoma macropomum* gills after baths with *Astrocaryum aculeatum* fixed oil solid lipid nanoparticles (250 mg/L), and efficacy.

**Treatments**	**Prevalence (%)**	**Mean Abundance**	**Efficacy (%)**
Culture tank water	100	20.1 ± 10.2^a^	-
Myristic acid + Tween 80	2.6	0.5 ± 1.1^a^	96.7
250 mg/L	47.7	1.4 ± 1.9^a^	0

Abundance data express mean ± standard deviation. Different letters in the same column indicate significant differences by the Dunn test (p<0.05).

### Blood parameters of *C. macropomum* after therapeutic baths with *A. aculeatum* fixed oil SLNs

In the fish subjected to baths with 250 mg/L of *A. aculeatum* oil SLNs and in control group fish exposed to myristic acid + Tween 80, the total number of erythrocytes and positive granular leukocytes (PAS-GL) decreased (p<0.05), while the total number of neutrophils increased (p<0.05) when compared to control group fish exposed only to water from the cultivation tank. The total number of lymphocytes decreased in the fish subjected to baths with 250 mg/L of *A. aculeatum* fixed oil SLNs when compared to fish of both control groups, while no significant changes (p > 0.05) were observed in the other blood parameters evaluated (Table 3).

**Table 3:** Blood parameters of *Colossoma macropomum* after therapeutic baths with *Astrocaryum aculeatum* fixed oil solid lipid nanoparticles (250 mg/L).

**Parameters**	**Water of culture**	**Myristic acid+ Tween 80**		**250 mg/L**	
Glucose (mg/dL)	96.1 ± 16.4^a^	90.4±21.7^a^	75.9 ± 21.2^a^	
Total protein (g/dL)	2.8 ± 0.7^a^	3.0 ± 0.5^a^	2.7 ± 0.3^a^	
Erythrocytes (x10^6^/μL)	3.0 ± 0.3^a^	2.7 ± 0.4^b^	2.6 ± 0.3^b^	
Hemoglobin (g/dL)	8.6 ± 0.8^a^	8.8 ± 1.2a	8.8 ± 0.9^a^	
Hematocrit (%)	27.8 ± 2.3^a^	28.9 ± 3.1^a^	26.9 ± 2.6^a^	
MCV (fL)	94.8 ± 13.5^a^	111.0 ± 22.9^a^	105.4 ± 14.2^a^	
MCHC (g/dL)	31.0 ± 3.0^a^	30.4 ± 3.1^a^	32.8 ± 2.9^a^	
Thrombocytes (μL)	78,887 ± 19,771^a^	73,601 ± 18,281^a^	48,533 ± 16,646^b^	
Leukocytes (μL)	21,257 ± 6,844^a^	18,351 ± 7,114^a^	16,354 ± 7,725^a^	
Lymphocytes (μL)	11,358 ± 4,308^a^	10,626 ± 14,310^a^	4,399 ± 2,289^c^	
Monocytes (μL)	5,929 ± 2,666^a^	5,962 ± 2,577^a^	4,483 ± 2,569^a^	
Neutrophils (μL)	3,402 ± 1,887^a^	6,419 ± 53,531^b^	7,499 ± 4,331^b^	
Eosinophils (μL)	134 ± 214^a^	8 ± 13^a^	43 ± 81^a^	
PAS-GL (μL)	358 ± 394^a^	4 ± 7^b^	49 ± 103^c^	

Data express mean ± standard deviation. Different letters, in the same row, indicate significant differences by the Dunn test (p<0.05). MCV: Mean Corpuscular Volume, MCHC: Mean Corpuscular Hemoglobin Concentration. PAS-GL: PAS-positive granular leukocytes.

### Histopathology of *C. macropomum* gills after therapeutic baths with *A. aculeatum* fixed oil SLNs

**Figure 1:**
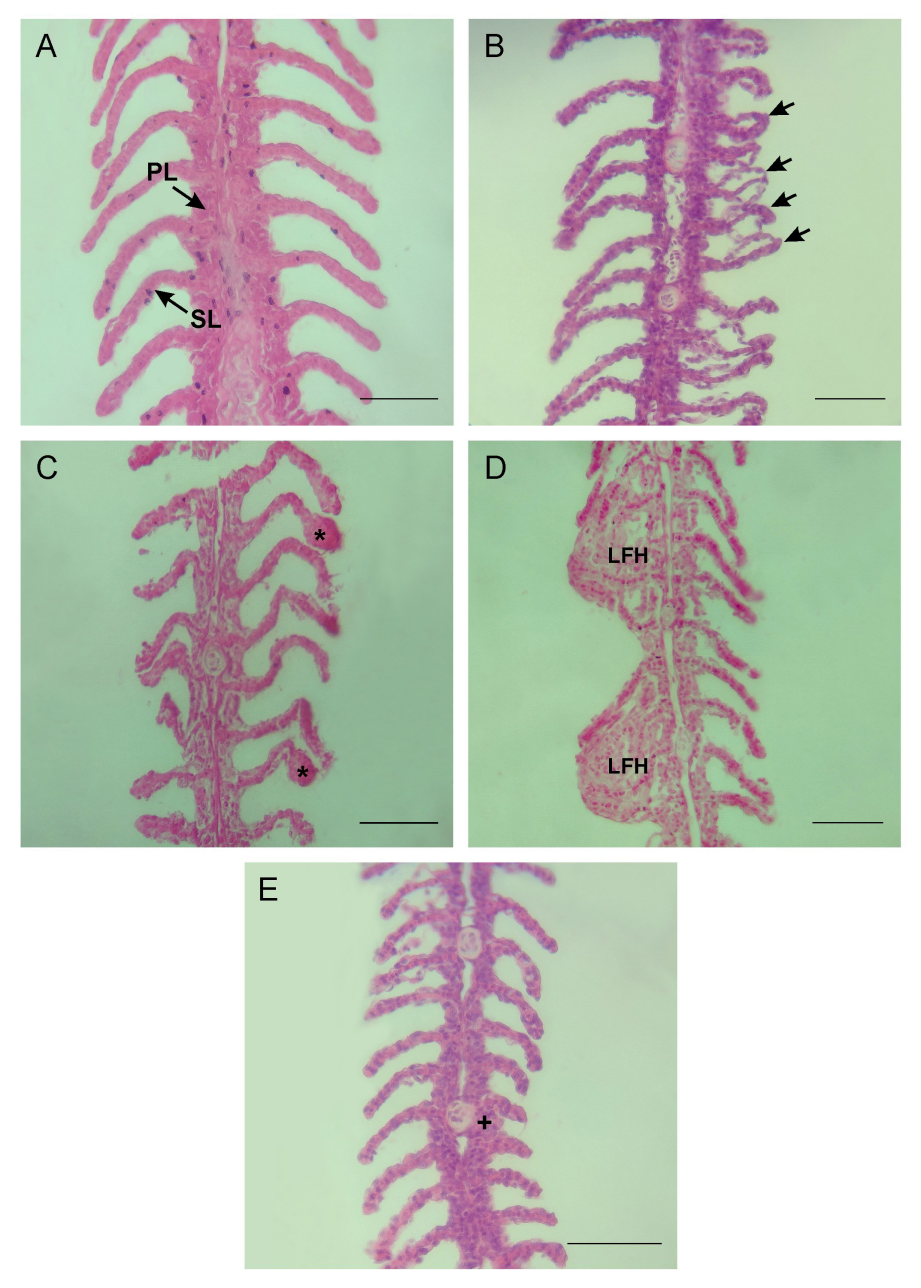
Histopathology of *Colossoma macropomum* gills exposed to solid lipid nanoparticles of *A. aculeatum* fixed oil*.* **A**. Gills with primary (PL) and secondary (SL) lamellae from fish exposed to water from the culture tank. **B**. Gill with filaments showing lesions such as detachment of the lamellar epithelium (arrows) in the control group of fish exposed to myristic acid + Tween 80. **C-E**. Gills of fish exposed to 250 mg/L of *A. aculeatum* fixed oil solid lipid nanoparticles. **C.** Branchial filament showing aneurysm lesions (asterisks). **D.** Branchial filament showing lamellar hyperplasia with fusion (LFH). **E**. Branchial filament with lamellar hyperplasia (cross). Staining: hematoxylin and eosin (HE) Scale bar = 20 µm.

The HAI and MAV of the fish gills subjected to five consecutive therapeutic baths with 250 mg/L of *A. aculeatum* fixed oil SLNs did not differ significantly (p< 0.05) from both the treatment controls. Histopathological analyses revealed mild to moderate alterations on *C. macropomum* gills submitted to both baths with 250 mg/L of *A. aculeatum* fixed oil SLNs and also controls. However, these changes did not affect gill function, as evidenced by the HAI index values (Table 4), which indicated the normal functioning of this respiratory organ. The main histopathological changes observed in the gills of fish exposed to the treatments and controls included similarly detachment of the lamellar epithelium, lamellar epithelial hyperplasia, lamellar hyperplasia with fusion, and aneurysm (Figure 1).

**Table 4:** Histopathological alteration index (HAI) and mean assessment values (MAV) for gills of *Colossoma macropomum* after therapeutic baths with *Astrocaryum aculeatum* fixed oil solid lipid nanoparticles (250 mg/L).

**Treatments**	**N**	**MAV**	**HAI**	**Severity of lesions according to HAI**
Culture tank water	9	1.7 ± 1.3^a^	1.9 ± 3.4^a^	Mild to moderates
Tween 80^®^ + myristic acid	9	2.4 ± 1.2^a^	2.7 ± 3.3^a^	Mild to moderates
250 mg/L	9	2.9 ± 1.8^a^	2.4 ± 3.6^a^	Mild to moderates

Data express mean ± standard deviation. Different letters, in the same column, indicate differences by the Dunn test (p<0.05). N: Sample number.

## Discussion

*Astrocaryumaculeatum* fixed oil usually presents a predominance of saturated fatty acids, but also contains carotenoids, i.e., β-carotene, phenolic compounds like gallic, caffeic, and chlorogenic acids, and phytosterols like campesterol, stigmasterol, and β-sitosterol (Yuyama et al., 2008; Jobim et al., 2014; Linhares et al., 2017; Ibiapina et al., 2022; Machado et al., 2022). The *A. aculeatum* fixed oil, used in this study, was composed predominantly by monounsaturated fatty acid like oleic acid (68%), with a smaller proportion of linoleic acid (2%). Similarly, Machado et al. (2022) stated that the fresh pulp of *A. aculeatum* possesses high levels of fatty acids (35-55%), primarily oleic acid (60-70%). In contrast, the major fatty acids identified in *A. aculeatum* fixed oil collected in the state of Rondônia were oleic acid (64.2%) and linoleic acid (11.0%) as major fatty acids (Linhares et al., 2017). Additionally, the acidity index of *A. aculeatum* fixed oil of this study was higher than the reported by Linhares et al. (2017) for the same oil, while the saponification index was lower. Since the saponification index is closely linked to the molecular mass of the triglycerides in *A. aculeatum* oil, the lower the index, the higher the molecular mass (Linhares et al., 2017).

The SLNs have a higher oil loading capacity than polymeric nanoparticles and other bilayer lipid particles. They also have many advantages, such as biocompatibility, biodegradability, and improved therapeutic efficacy. They easily disperse in the cell membranes of targeted organisms (Wissing et al., 2004; Mogahed et al., 2023; Nemati et al., 2024; Abdelkarim et al., 2025). However, zeta potential is an important parameter relating to the surface potential of these nanoparticle droplets, and high values are associated with stable systems. The polydispersity index is a parameter associated with the homogeneity of particle populations and results. Desirable values are within the range of 0 (monodispersed) to 0.500 (relatively broad distribution), and acceptable values are below 0.700 (Wissing et al., 2004; Valentim et al., 2018; Oliveira et al., 2022). The SLNs of *A. aculeatum* oil had a zeta potential and polydispersity index close to the values reported for *Bixa orellana* oil SNLs (Oliveira et al., 2022), though the particle size was larger. The PDI value of the *A. aculeatum* fixed oil SLNs used in this indicated a relatively narrow particle size distribution. The droplet size and polydispersity suggest the kinetic stability of this nanoformulation, but the size found was satisfactory.

During the tolerance trials, we observed behavioral changes in *C. macropomum* associated with stress and mortality at the highest concentrations (500 and 1000 mg/L of oil SLN) after 8 h of exposure. Fish submitted to baths with 250 mg/L of *A. aculeatum* oil SLNs displayed similar changes, but no mortality occurred during this exposure time. Similar behavioral changes and no mortality of *C. macropomum* exposed to nanoemulsion with *Copaifera reticulata* Ducke oleoresin and this oleoresin non-nanoform (Malheiros et al., 2020), as well as for this same fish when exposed to *Carapa guianensis* (Aublet) Steudel fixed oil (Malheiros et al., 2023); while this nanoemulsion with *C. reticulata* oils was less toxic than the oleoresin in its non-nanoform. Therefore, our founding’s showed that 250 mg/L of *A. aculeatum* fixed oil SLNs were safer for *C. macropomum*. However, studies have reported that while crude extract of *A. aculeatum* did not induce toxicity for female rats exposed to low concentration in a single or repeated dose, it was safe for male rats only when administered repeatedly in low doses (Guex et al., 2020).

We found that the therapeutic baths with *A. aculeatum* fixed oil SLNs did not exhibit efficacy anti-*A. spathulatus, N. janauachensis*, and *M. boegeri* on *C. macropomum* gills, as expected; because the parasiticidal action was displayed by the surfactants myristic acid and Tween 80 used in preparation of SLNs, which had lower mean abundance than group control with cultivation tank water. Similarly, studies by Steverding et al. (2005) have also shown that *Melaleuca alternifolia* essential oil has negative effect on species of *Gyrodactylus* in *Gasterosteus aculeatus*. The parasiticidal action of this oil primarily occurred in the presence of the Tween 80, the solubilizing agent of essential, considering in the absence of this solvent this the *M. alternifolia* oil exhibited little effect on these parasites. Baths of *C. macropomum* with 500 mg/L de *P. macroloba* oil SLNs were also ineffective against *A. spathulatus, N. janauachensis*, and *M. boegeri* due to the parasiticidal action of the surfactants myristic acid and Tween 80 (Malheiros et al., 2025). Other similar studies have also reported antiparasitic activity of solvents as ethylic alcohol, iso-propyl alcohol, Tween 80, Tween 20 and dimethyl sulfoxide (DMSO) when used as solubilizing for essential oils and fixed oils (Tavares-Dias, 2018; Malheiros et al., 2023), albeit lowest than of these studies. These findings show therefore the potential of myristic acid and Tween 80 as an effective treatment of these parasites of *C. macropomum* gills. It was pustuled that due to myristic acid pharmacological properties, this presents antiparasitic activity (Rehmana et al., 2017; Javid et al., 2020), although this activity has been not investigated at present moment. Interestingly, myristic acid plus Tween 80 displayed parasiticidal activity against *A. spathulatus*, *N. janauachensis*, and *M. boegeri* of *C. macropomum,* although they are non-toxic surfactant widely used as an emulsifier for oils or nanoformulations. In contrast to our findings, bactericidal and antifungal activity of the ethanolic extract of the pulp and peel of *A. aculeatum* has been demonstrated by Jobim et al. (2014).

On *C. macropomum* gills exposed to baths with 250 mg/L of *A. aculeatum* fixed oil SLNs and both control groups (water from cultivation tank and myristic acid + Tween 80), the main lesions found were detachment and hyperplasia of the lamellar epithelium, lamellar hyperplasia with fusion, and aneurysm, which did not compromise the function of the respiratory organ Similar alterations have been reported for *C. macropomum* after baths with 500 mg/L de *P. macroloba* oil SLNs (Malheiros et al., 2025). Therefore, these branchial alterations may be a strategy of the respiratory organ in response to infections by *A. spathulatus*, *N. janauachensis,* and *M. boegeri*. Similar damages in the gills of *C. macropomum* infected by these same parasites were found by Tavares-Dias et al. (2021). Sajiri et al. (2025) also reported gill alterations in *Silurus glanis* Linnaeus, 1758 caused by *Thaparocleidus vistulensis* (Siwak, 1932), such as epithelial hyperplasia leading to lamellar fusion and subsequent extravasated erythrocytes, forming club-like structures, and the presence of eosinophilic granular cells in the epithelium. These results indicate that these ectoparasitic monopisthocotylans cause severe damage to the gill filaments of their host fish. However, similar alterations have been reported in the gills of *C. macropomum* due to therapeutic baths with *C. reticulata* oleoresin (Malheiros et al., 2022) and *C. guianensis* fixed oil (Malheiros et al., 2023); indicating therefore that phytotherapeutic agents may also cause structural effects in this tissue.

On *C. macropomum*, therapeutic baths with 250 mg/L of *A. aculeatum* fixed oil SLNs decreased the total number of lymphocytes; while both treatments with of *A. aculeatum* fixed oil SLNs and control with myristic acid + Tween 80 decreased the total number of erythrocytes and PAS-GL, and increased the total number of neutrophils. Baths of *C. macropomum* with 500 mg/L de *P. macroloba* oil SLNs only increased plasma total protein levels (Malheiros et al., 2025). In contrast, *A. aculeatum* crude extract did not affect total erythrocyte or leukocyte parameters on rats (Guex et al., 2020). However, these blood alterations on *C. macropomum* were few compared to those reported for therapeutic baths with 500 mg/L of *C. guianensis* fixed oil in same this fish that caused increase in plasma levels of glucose and total protein, as well as an increase in the total number of erythrocytes, thrombocytes, leukocytes, lymphocytes, monocytes and eosinophils, and a decrease in MCV. Both leukocytosis and erythrocytosis usually occur in cases of infections and/or exposure to irritating substances (Ranzani-Paiva et al., 2013; Malheiros et al., 2023), due to the stimulated production of these blood cells on hematopoietic organs. Piscine erythrocytes are cells responsible for transporting oxygen and may respond to exposure to various toxic substances, while thrombocytes primarily have a homeostatic function and a secondary role in the phagocytic process. PAS-GLs are granulocytes without a precisely defined function; however, they occur in greater numbers in parasitized fish or those subjected to induced inflammatory processes (Ranzani-Paiva et al., 2013). Therefore, this indicates immune functions that need to be yet properly classified.

## Conclusions

The *A. aculeatum* fixed oil, which contains oleic acid as the primary fatty acid, was used to prepare SLNs. *Colossoma macropomum* exhibited tolerance to these SLNs in a dose-dependent manner. Although therapeutic baths with *A. aculeatum* fixed oil SLNs do not exhibit efficacy against *A. spathulatus, N. janauachensis,* and *M. boegeri* of *C. macropomum* gills, they do not cause any histopathological damage or physiological changes on fish.

## Data Availability

Data will be made available upon request.

## References

[B001] Abdelkarim EA, Elsamahy T, El Bayomi RM, Hussein MA, Darwish IA, El‑tahlawy AS (2025). Nanoparticle‑driven aquaculture: transforming disease management and boosting sustainable fish farming practices. Aquacult Int.

[B002] Ahmed MT, Ali MS, Ahamed T, Sharmin S, Haq M (2024). Exploring the aspects of the application of nanotechnology system in aquaculture: a systematic review. Aquacult Int.

[B003] Alves CMG, Baia RRJ, Farias VA, Farias MA, Souza FLS, Videira MN (2024). Essential oil of *Piper hispidum* (Piperaceae) has efficacy against monogeneans, and effects on hematology and gill histology of *Colossoma macropomum.*. Rev Bras Parasitol Vet.

[B004] Brito BD, Souza Marinho VH, Ferreira IM, Tavares-Dias M (2026). Phytotherapeutics for parasite control in global fish aquaculture: a review of anti-monogenean agents and their mechanisms. Aquat Living Resour.

[B005] Bush AO, Lafferty KD, Lotz JM, Shostak AW (1997). Parasitology meets ecology on its own terms: Margolis et al. revisited. J Parasitol.

[B006] Eiras JC, Takemoto RM, Pavanelli GC (2006). Métodos de estudos e técnicas laboratoriais em parasitologia de peixes..

[B007] FAO (2024). The state of world fisheries and aquaculture 2024 – Blue Transformation in action..

[B008] Guex CG, Cassanego GB, Dornelles RC, Casoti R, Engelmann AM, Somacal S (2020). Tucumã (*Astrocaryum aculeatum*) extract: phytochemical characterization, acute and subacute oral toxicity studies in *Wistar* rats. Drug Chem Toxicol.

[B009] Ibiapina A, Gualberto LS, Dias BB, Freitas BCB, Martins GAS, Melo-Filho AA (2022). Essential and fixed oils from Amazonian fruits: proprieties and applications. Crit Rev Food Sci Nutr.

[B010] IAL (2008). Métodos físico-químicos para análise de alimentos..

[B011] Javid S, Purohit MN, Kumar HY, Ramya K, Mithuna NFA, Salahuddin MD (2020). Semisynthesis of myristic acid derivatives and their biological activities: a critical insight. J Biol Act Prod Nat.

[B012] Jobim ML, Santos RCV, Alves CFS, Oliveira RM, Mostardeiro C, Sagrillo MR (2014). Antimicrobial activity of Amazon *Astrocaryum aculeatum* extracts and its association to oxidative metabolism. Microbiol Res.

[B013] Kumari P, Kumar S, Raman RP, Brahmchari RK (2024). Nanotechnology: an avenue for combating fish parasites in aquaculture system. Vet Parasitol.

[B014] Linhares BM, Costa AMDC, Abreu HDF, Melo ACGR, Ribeiro PRE, Montero IF (2017). Fatty acids profile, physicalchemical properties and minerals with quantify indicator of *Astrocaryum aculeatum* pulp oil. J Agric Sci (Toronto).

[B015] Lopes SQ, Holanda FH, Jimenez DEQ, Nascimento LAS, Oliveira AN, Ferreira IM (2021). Use of Oxone^®^ as a potential catalyst in biodiesel production from palm fatty acid distillate (PFAD). Catal Lett.

[B016] Machado APF, Nascimento RP, Alves MR, Reguengo LM, Marostica MR (2022). Brazilian tucumã-do-Amazonas (*Astrocaryum aculeatum*) and tucumã-do-Pará (*Astrocaryum vulgare*) fruits: bioactive composition, health benefits, and technological potential. Food Res Int.

[B017] Malheiros DF, Sarquis IR, Ferreira IM, Mathews PD, Mertins O, Tavares-Dias M (2020). Nanoemulsions with oleoresin of *Copaifera reticulata* (Leguminosae) improve anthelmintic efficacy in the control of monogenean parasites when compared to oleoresin without nanoformulation. J Fish Dis.

[B018] Malheiros DF, Videira MN, Ferreira IM, Tavares-Dias M (2022). Anthelmintic efficacy of *Copaifera reticulata* oleoresin in the control of monogeneans and haematological and histopathological effects on *Colossoma macropomum.*. Aquacult Res.

[B019] Malheiros DF, Videira MN, Carvalho AA, Salomão CB, Ferreira IM, Canuto KM (2023). Efficacy of *Carapa guianensis* oil (Meliaceae) against monogeneans infestations: a potential antiparasitic for *Colossoma macropomum* and its effects in hematology and histopathology of gills. Rev Bras Parasitol Vet.

[B020] Malheiros DF, Martins MF, Marinho VHS, Ferreira IM, Yoshioka ETO, Magalhães HCR (2025). Anti-dactylogyridean efficacy of solid lipid nanoparticles of *Pentaclethra macroloba* oleoresin in *Colossoma macropomum*, with hematology and gill histopathology assessment. Braz J Biol.

[B021] Marinho VHS, Holanda FH, Araújo IF, Jimenez DEQ, Pereira RR, Porto ALM (2023). Nanoparticles from silk fibroin and Amazon oils: potential larvicidal activity and oviposition deterrence against *Aedes aegypti.*. Ind Crops Prod.

[B022] Mogahed NMFH, El-Temsahy MM, Abou-El-Naga IF, Makled S, Sheta E, Eman S (2023). Loading praziquantel within solid lipid nanoparticles improved its schistosomicidal efficacy against the juvenile stage. Exp Parasitol.

[B023] Nemati S, Mottaghi M, Karami P, Mirjalali H (2024). Development of solid lipid nanoparticles‑loaded drugs in parasitic diseases. Discov Nano.

[B024] Oliveira SSC, Sarmento ES, Marinho VH, Pereira RR, Fonseca LP, Ferreira IM (2022). Green extraction of annatto seed oily extract and its use as a pharmaceutical material for the production of lipid nanoparticles. Molecules.

[B025] Poleksić V, Mitrović-Tutundžić V, Muller R, Lloyd R (1994). Sublethal and chronic effects of pollutants on freshwater fish..

[B026] Qu S, Liu J, Wu Z, Li J, Li P, Wang G (2023). Nanocomplexation is a promising strategy to enhance the solubility and anti-*Ichthyophthirius multifiliis* activity of magnolol. Aquaculture.

[B027] Ranzani-Paiva MJT, Pádua SB, Tavares-Dias M, Egami MI (2013). Métodos para análise hematológica em peixes..

[B028] Rehmana M, Arshad A, Asadullah Madni MA (2017). Nanoformulated myristic acid for antimicrobial applications. GPSR.

[B029] Sajiri WMHW, Székely C, Molnár K, Buchmann K, Sellyei B (2025). Pathological effects of *Thaparocleidus vistulensis* (Siwak, 1932) infection on the gills of *Silurus glanis* Linnaeus, 1758. Acta Vet Hung.

[B030] Sarquis IR, Sarquis RSFR, Marinho VHS, Neves FB, Araújo IF, Damasceno LF (2020). Carapa guianensis Aubl. (Meliaceae) oil associated with silk fibroin, as alternative to traditional surfactants, and active against larvae of the vector Aedes aegypti.. Ind Crops Prod.

[B031] Schwaiger J, Wanke R, Adam S, Pawert M, Honnen W, Triebskorn R (1997). The use of histopathological indicators to evaluate contaminant-related stress in fish. J Aquat Ecosyst Stress Recovery.

[B032] Shinn AP, Pratoomyot J, Bron JE, Paladini G, Brooker EE, Brooker AJ (2015). Economic costs of protistan and metazoan parasites to global mariculture. Parasitology.

[B033] Steverding D, Morgan E, Tkaczynski P, Walder F, Tinsley R (2005). Effect of Australian tea tree oil on *Gyrodactylus* spp. infection of the three-spined stickleback *Gasterosteus aculeatus.*. Dis Aquat Organ.

[B034] Tavares-Dias M, Ferreira GV, Videira MN (2021). Histopathological alterations caused by monogenean parasites the gills of tambaqui *Colossoma macropomum* (Serrasalmidae). Semina: Ciênc Agrár.

[B035] Tavares-Dias M, Martins ML (2017). An overall estimation of losses caused by diseases in the Brazilian fish farms. J Parasit Dis.

[B036] Tavares-Dias M (2018). Current knowledge on use of essential oils as alternative treatment against fish parasites. Aquat Living Resour.

[B037] Valentim DSS, Duarte JL, Oliveira AEMFM, Cruz RAS, Carvalho JCT, Solans C (2018). Effects of a nanoemulsion with *Copaifera officinalis* oleoresin against monogenean parasites of *Colossoma macropomum*: a Neotropical Serrasalmidae. J Fish Dis.

[B038] Wang GX, Zhou Z, Cheng C, Yao JY, Yang ZW (2008). Osthol and isopimpinellin from *Fructus cnidii* for the control of *Dactylogyrus intermedius* in *Carassius auratus.*. Vet Parasitol.

[B039] Wissing SA, Kayser O, Müller RH (2004). Solid lipid nanoparticles for parenteral drug delivery. Adv Drug Deliv Rev.

[B040] Yuyama LKO, Maeda RN, Pantoja L, Aguiar JPL, Marinho HA (2008). Processamento e avaliação da vida-de-prateleira do tucumã (*Astrocaryum aculeatum* Meyer) desidratado e pulverizado. Food Sci Technol (Campinas).

[B041] Zar JH (2010). Biostatistical analysis..

